# Cost-effectiveness of endovascular treatment after 6–24 h in ischaemic stroke patients with collateral flow on CT-angiography: A model-based economic evaluation of the MR CLEAN-LATE trial

**DOI:** 10.1177/23969873231220464

**Published:** 2023-12-28

**Authors:** Florentina ME Pinckaers, Silvia MAA Evers, Susanne GH Olthuis, Hieronymus D Boogaarts, Alida A Postma, Robert J van Oostenbrugge, Wim H van Zwam, Janneke PC Grutters

**Affiliations:** 1 Department of Radiology and Nuclear Medicine, Maastricht University Medical Centre, Maastricht, The Netherlands; 2 School for Cardiovascular Diseases (CARIM), Maastricht University, Maastricht, The Netherlands; 3 Care and Public Health Research Institute (CAPHRI), Maastricht University, Maastricht, The Netherlands; 4 Department of Health Services Research, Maastricht University, Maastricht, The Netherlands; 5 Centre of Economic Evaluation and Machine Learning, Trimbos Institute, Utrecht, The Netherlands; 6 Department of Neurology, Maastricht University Medical Centre, Maastricht, The Netherlands; 7 Department of Neurosurgery, Radboudumc, Nijmegen, The Netherlands; 8 School for Mental Health and Neuroscience (MHENS), Maastricht University, Maastricht, The Netherlands; 9 Department for Health Evidence, Radboudumc, Nijmegen, The Netherlands

**Keywords:** Ischaemic stroke, thrombectomy, cost-effectiveness analysis, late window, collateral circulation

## Abstract

**Background:**

The MR CLEAN-LATE trial has shown that patient selection for endovascular treatment (EVT) in the late window (6–24 h after onset or last-seen-well) based on the presence of collateral flow on CT-angiography is safe and effective. We aimed to assess the cost-effectiveness of late-window collateral-based EVT-selection compared to best medical management (BMM) over a lifetime horizon (until 95 years of age).

**Materials and Methods:**

A model-based economic evaluation was performed from a societal perspective in The Netherlands. A decision tree was combined with a state-transition (Markov) model. Health states were defined by the modified Rankin Scale (mRS). Initial probabilities at 3-months post-stroke were based on MR CLEAN-LATE data. Transition probabilities were derived from previous literature. Information on short- and long-term resource use and utilities was obtained from a study using MR CLEAN-LATE and cross-sectional data. All costs are expressed in 2022 euros. Costs and quality-adjusted life years (QALYs) were discounted at a rate of 4% and 1.5%, respectively. The effect of parameter uncertainty was assessed using probabilistic sensitivity analysis (PSA).

**Results:**

On average, the EVT strategy cost €159,592 (95% CI: €140,830–€180,154) and generated 3.46 QALYs (95% CI: 3.04–3.90) per patient, whereas the costs and QALYs associated with BMM were €149,935 (95% CI: €130,841–€171,776) and 2.88 (95% CI: 2.48–3.29), respectively. The incremental cost-effectiveness ratio per QALY and the incremental net monetary benefit were €16,442 and €19,710, respectively. At a cost-effectiveness threshold of €50,000/QALY, EVT was cost-effective in 87% of replications.

**Discussion and Conclusion:**

Collateral-based selection for late-window EVT is likely cost-effective from a societal perspective in The Netherlands.

## Graphical abstract

**Graphical abstract:**
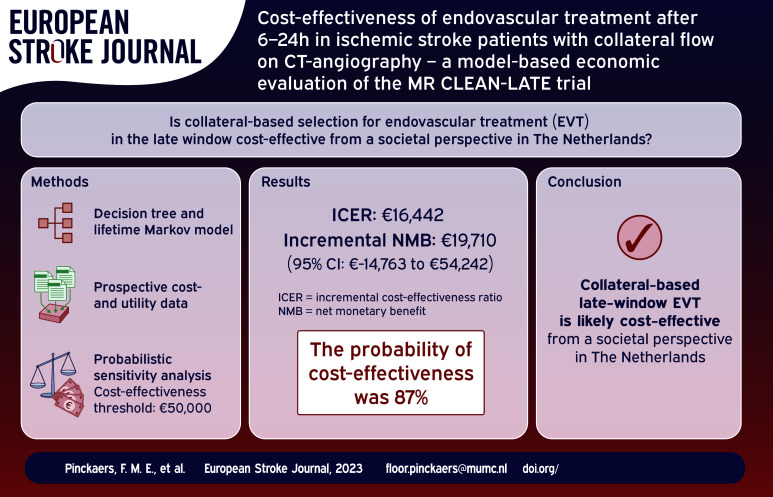

## Introduction

The MR CLEAN-LATE trial has recently shown the efficacy of collateral-based selection for endovascular treatment (EVT) in the late window (6–24 h after onset or last-seen-well) on functional outcomes at 90 days post-stroke, identifying an additional patient population benefitting from acute stroke treatment (adjusted common odds ratio for shift towards better outcomes: 1.67 [95% CI 1.20–2.32]).^1^ Though these results provide new opportunities for improving patient outcomes, the costs associated with EVT can be high. As budget constraints and increasing expenditures are challenging many healthcare systems, it is crucial to determine whether the observed gains in functional outcome in the MR CLEAN-LATE trial also translate into long-term societal benefits.

Previous studies have evaluated the cost-effectiveness of CTP-based late-window treatment selection.^2–5^ The aim of the present study was to evaluate the cost-effectiveness of collateral-based EVT in the late window compared to best medical management (BMM) from a societal perspective in The Netherlands using a model-based approach. These results may inform policy makers on resource allocation and reimbursement.

## Methods

This manuscript was prepared according to the Consolidated health economic evaluation reporting standards (CHEERS) guidelines, the ISPOR-SMDM Modelling good research practices guidelines, and the Dutch guidelines on economic evaluations.^6–8^

### Patient population

This economic evaluation was based on data of the MR CLEAN-LATE trial (Multicenter Randomized Clinical Trial of Endovascular Treatment of Acute Ischaemic Stroke in The Netherlands for LATE arrivals; International Standard Randomised Controlled Trial Number: ISRCTN19922220).^1^ The MR CLEAN-LATE aimed to assess the safety and efficacy of collateral flow-based selection for endovascular treatment (EVT) for acute ischaemic stroke in the late window (6–24 h after onset or last-seen-well). Eighteen stroke intervention centres in The Netherlands participated. Trial inclusion criteria included a proximal occlusion in the anterior circulation (i.e. distal intracranial internal carotid artery, or the first or second segment of the middle cerebral artery), possibility of EVT commencement at 6–24 h from symptom onset or last-seen well, and at least some collateral flow on CT angiography. Exclusion criteria included the presence of a favourable clinical and imaging profile similar to the study populations included in the DAWN- and DEFUSE-3 trials, as these patients were eligible for EVT treatment according to Dutch guidelines.^9,10^ Patients were randomised 1:1 to the EVT or BMM arm.

### Study design

Methods of the economic evaluation were agreed on prior to analysis commencement. A lifetime health economic model was constructed from a societal perspective in The Netherlands. Effects were expressed in quality-adjusted life year (QALYs), which were calculated by multiplying the utility value (a measure of health-related quality of life) of a health state by the years lived in that state. Based on the median age of the MR CLEAN-LATE cohort, the starting age of the model population was 74 years. Model input parameters on costs and effects were based on data collected in parallel to the trial proceedings. Costs and effects after 1 year post-stroke were discounted with 4% and 1.5%, respectively, in accordance with Dutch guidelines.^8^

### Model structure

**Figure 1.**
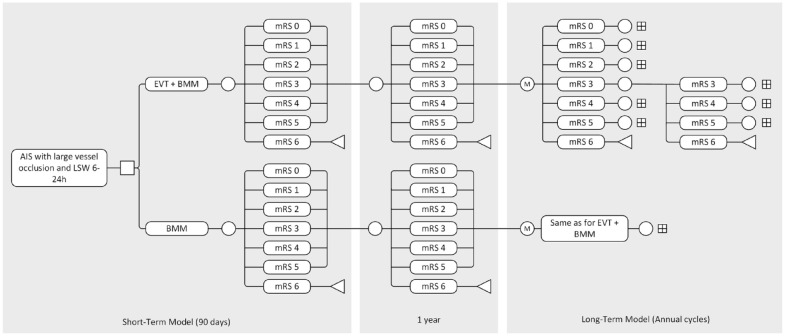
Model structure. AIS: acute ischaemic stroke; BMM: best medical management; EVT: endovascular treatment; mRS: modified Rankin Scale; LSW: time last-seen-well. □: decision node; ○: chance node; M: Markov node; v: end node.

The model structure is described in Figure 1. A short-term decision tree was combined with a long-term time-dependent cohort state-transition (Markov) model with a lifetime horizon (up to 95 years of age). Health states were based on the modified Rankin Scale (mRS) score. It was assumed that patients could move freely across health states (i.e. patients could improve in mRS, remain stable, deteriorate in mRS, or die) between 3-months and 1-year post-stroke. From here, patients entered a Markov model with yearly cycles, in which patients could only remain in the same health state, deteriorate, or die.

### Model parameters

#### Transition probabilities

The mRS distribution at 3-months post-stroke for the two treatment arms was derived from the MR CLEAN-LATE trial. Transition probabilities after 3-months were based on data from the Oxford Vascular study describing mRS-score changes between 3-months and 1-year-, and between 1-year and 5-years post-stroke.^11^ Transition rates from the source data were converted to probabilities appropriate to cycle length.^12^ For transitions beyond 5-years post-stroke, the mortality probability was inflated using an age-dependent mortality penalty.^13^ Base-case values for the initial mRS distribution and transition probabilities may be found in Supplemental Appendix I. A graphical presentation of the cohort trace may be found in Supplemental Appendix II.

#### Costs

A pooled dataset, containing MR CLEAN-LATE and cross-sectional follow-up data at 3-months, 1-year and 2-years post-stroke, was used to derive mean cost estimates for mRS 0–5 by means of non-parametric bootstrapping combined with multiple imputation. Comprehensive methods and results have been described previously.^14^ Costs (in euros, calculated for the year 2022) were assessed from a societal perspective and included professional home care, informal care, transportation, mobility and home aids, and productivity losses. Assessed healthcare costs included ambulance transfers, emergency room assessment, intravenous thrombolysis and endovascular treatment, (intensive care) ward admission days, in-hospital cerebral imaging and post-discharge healthcare consumption.

For patients who were deceased at 3-months post-stroke, a mean cost estimate was derived using non-parametric bootstrapping with 2000 replications using the following data. Healthcare costs were derived from MR CLEAN-LATE records on hospitalisation, discharge location, and date of death. Considering non-healthcare costs, it was assumed that paid and unpaid productivity losses for deceased patients of working age were equal to the mean estimate of patients with an mRS-score of 5; for patients older than working age, only unpaid productivity loss estimates were used. Fourteen percent of the deceased population met the criterium of being of ‘working age’, which was defined as an age of 64 years or younger. No other non-healthcare costs were included in the cost estimates for patients deceased within 3-months post-stroke. Information on the applied unit costs may be found in the previously mentioned paper.^14^

Base-case values for costs are described in Supplemental Appendix I.

#### Utilities

Utility values per mRS were derived from the previously mentioned pooled dataset.^14^ EQ-5D-5L-responses were converted into utilities using a Dutch value-set based on composite time trade-off.^15^ Mean utility estimates per mRS were derived using non-parametric bootstrapping combined with multiple imputation.

Base-case values for utilities are shown in Supplemental Appendix I.

### Cost-effectiveness analysis

Total costs and effects per patient were calculated for each strategy, as were the incremental costs and effects. The incremental cost-effectiveness ratio (ICER) and incremental net monetary benefit (iNMB) were assessed (formulas may be found in Supplemental Appendix III). Considering the cost-effectiveness threshold, current Dutch guidelines suggest that this should be dependent on the disease burden in the population of interest, and calculated by assessing the proportional shortfall of prospective health.^16^ Using this method, the iMTA Disease Burden Calculator (iDBC) yielded a threshold of €50,000/QALY for our population (85% likelihood of applicability; a threshold of €80,000/QALY had a 15% likelihood of applicability).^17^

A probabilistic sensitivity analysis (PSA) with 10,000 simulations was performed in order to assess how parameter uncertainty affected our results. A distribution was assigned to each parameter according to the level of uncertainty regarding its deterministic value (Supplemental Appendix I). For the initial cohort mRS-distribution and transition probabilities, a Dirichlet distribution was used. Costs were varied using a gamma distribution. Utility samples were drawn from a gamma distribution on disutility (1 - utility), as this allowed for the occurrence of negative utilities (i.e. health states worse than death). From the simulations, 95% confidence intervals (CI) were calculated using the percentile method. Results of the PSA were presented on a cost-effectiveness plane (scatter plot) and a cost-effectiveness acceptability curve.

A one-way sensitivity analysis was performed in order to assess the effect of changing a single parameter on the iNMB. The evaluated parameters were: the initial mRS distribution of the EVT strategy; costs; utilities. The minimum and maximum value of each parameter was derived from the simulated PSA range. As the initial cohort distribution was varied using a Dirichlet distribution, the mRS distribution samples with the lowest and highest mortality probability were used.

Analyses were performed in R version 4.3.0.^18^

## Results

**Figure 2.**
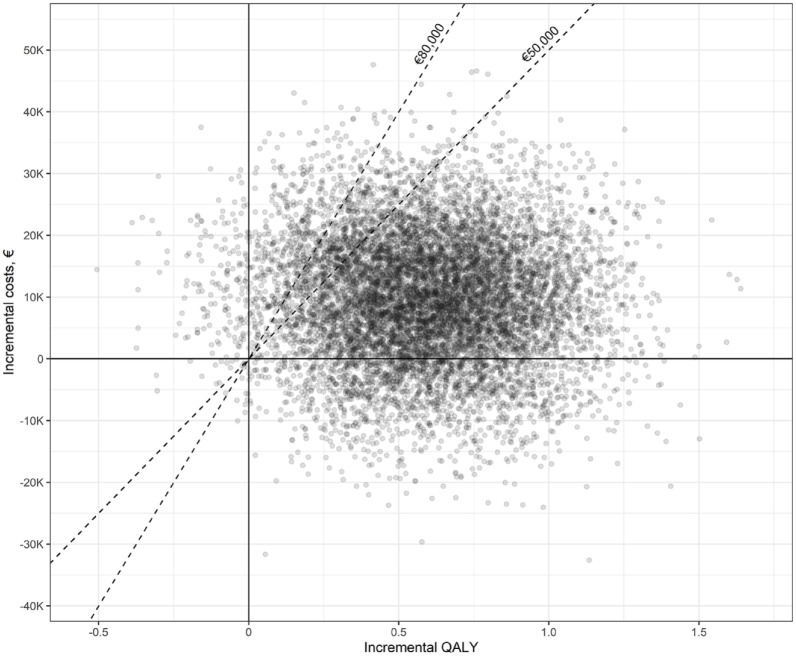
Results of the probabilistic sensitivity analysis (societal perspective). The dashed lines indicate the cost-effectiveness thresholds of €50,000 and €80,000 per QALY.

**Figure 3.**
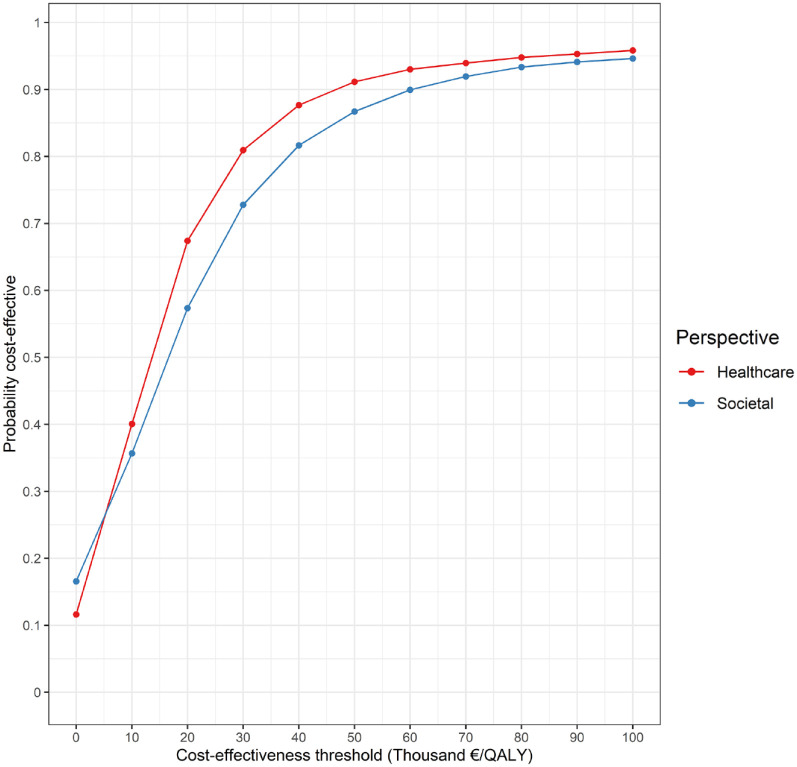
Cost-effectiveness acceptability curve. At a cost-effectiveness threshold of €50,000/QALY, EVT was cost-effective in 87% of replications from a societal perspective, and in 91% of replications from a healthcare perspective.

From a societal perspective, the EVT strategy cost €159,592 (95% CI: €140,830–€180,154) and generated 3.46 QALYs (95% CI: 3.04–3.90) per patient, whereas the costs and QALYs associated with BMM were €149,935 (95% CI: €130,841–€171,776) and 2.88 (95% CI: 2.48–3.29), respectively (Table 1). The mean ICER and iNMB were €16,442 and €19,710, respectively (Table 1). A scatter plot of the PSA-derived ICERs is depicted in Figure 2. At the €50,000 threshold, the probability of EVT being cost-effective was 87% (Figure 3).

**Table 1.** Results from a societal perspective.

Strategy	Costs, € (95% CI)	Effects, QALY (95% CI)	Incremental costs, € (95% CI)	Incremental effects, QALY (95% CI)	ICER, €/QALY	Incremental NMB, € (95% CI)^a^
Base case						
BMM	149,856	2.87	-	-	-	-
EVT	159,684	3.46	9827	0.59	16,645	19,694
PSA						
BMM	149,935 (130,841 to 171,776)	2.88 (2.48 to 3.29)	-	-	-	-
EVT	159,592 (140,830 to 180,154)	3.46 (3.04 to 3.90)	9657 (−10,252 to 29,410)	0.59 (0.03 to 1.14)	16,442	19,710 (−14,763 to 54,242)

BMM: best medical management; EVT: endovascular treatment; ICER: incremental cost-effectiveness ratio; NMB: net monetary benefit; PSA: probabilistic sensitivity analysis; QALY: quality-adjusted life year.

a NMB at a cost-effectiveness threshold of €50,000/QALY.

Results from a healthcare perspective are presented in Table 2. The costs and QALYs per patient were, respectively, €100,103 (95% CI: €86,997 to €115,379) and 3.46 QALYs (95% CI: 3.04–3.90) for the EVT strategy, and €92,414 (95% CI: €78,464–€108,120) and 2.88 QALYs (95% CI: 2.48–3.29) for BMM. The mean ICER and iNMB were €13,091 and €21,678, respectively. A scatter plot of the PSA-derived ICERs from a healthcare perspective may be found in Supplemental Appendix IV. At the €50,000 threshold, EVT was cost-effective in 91% of replications (Figure 3).

**Table 2.** Results from a healthcare perspective.

Strategy	Costs, € (95% CI)	Effects, QALY (95% CI)	Incremental costs, € (95% CI)	Incremental effects, QALY (95% CI)	ICER, €/QALY	Incremental NMB, € (95% CI)^a^
Base case						
BMM	92,332	2.87	-	-	-	-
EVT	100,132	3.46	7800	0.59	13,211	21,721
PSA						
BMM	92,414 (78,464 to 108,120)	2.88 (2.48 to 3.29)	-	-	-	-
EVT	100,103 (86,997 to 115,379)	3.46 (3.04 to 3.90)	7,689 (−5519 to 20,283)	0.59 (0.03 to 1.14)	13,091	21,678 (−9926 to 52,907)

BMM: best medical management; EVT: endovascular treatment; ICER: incremental cost-effectiveness ratio; NMB: net monetary benefit; PSA: probabilistic sensitivity analysis; QALY: quality-adjusted life year.

a NMB at a cost-effectiveness threshold of €50,000/QALY.

A tornado diagram of the one-way sensitivity analysis from a societal perspective may be found in Supplemental Appendix V. The initial mRS distribution of the EVT strategy had the highest impact on the iNMB, followed by the cost input parameters for the first 3 months post-stroke.

Undiscounted PSA results may be found in Supplemental Appendix VI. From a societal perspective, the mean ICER and iNMB were €17,607 and €26,499, respectively. From a healthcare perspective, undiscounted analyses resulted in a mean ICER and iNMB of €12,201 and €30,992, respectively.

## Discussion

In this study, we aimed to assess the cost-effectiveness of late-window EVT in patients selected based on the presence of collaterals on CT-angiography, which was recently proven effective in the MR CLEAN-LATE trial.^1^ Our main finding was that EVT, compared to BMM, is likely cost-effective from a societal perspective in The Netherlands.

Previous cost-effectiveness analyses on late-window EVT were primarily informed by the results of the DAWN- and DEFUSE-3 trials, and reported healthcare payer perspective ICERs ranging from −€21,504 to €31,233.^2–5^ Though our current study was informed by the results of the MR CLEAN-LATE trial – in which patients with a favourable prognosis based on the DAWN- and DEFUSE-3 results were excluded, likely resulting in a lower treatment effect estimate – the healthcare perspective ICER found in the present study (€13,091 per QALY) lies well within the ICER-range reported in previous literature. From a societal perspective, we report an ICER of €16,442, and our PSA indicates that EVT was cost-effective in 87% of replications at a cost-effectiveness threshold of €50,000. These results provide clear directions on the implementation and reimbursement of collateral-based late-window EVT.

We would like to highlight a few important differences between the model used in the present study and the models used in previous literature. First of all, we draw attention to the modelling of mRS deterioration. Most previous studies only allowed for a deterioration in mRS score as a result of recurrent stroke, which only occurs in a relatively small proportion of patients every model cycle.^2–4^ This may result in an excess of patients retaining a good mRS-score, generating QALYs at a relatively low cost and consequently biasing model results. In the present study, mRS deterioration took place irrespective of recurrent stroke, which allowed for faster changes in the mRS distribution of the cohort (Supplemental Appendix II). In our opinion, this better reflects the true development in functional outcome in a cohort of elderly stroke patients. Second, instead of relying on previous literature for cost- and utility input parameters, we prospectively collected (long-term) data in parallel to the MR CLEAN-LATE trial’s proceedings. Considering utilities, this has the advantage that our utility estimates were based on the EQ-5D-5L instead of the EQ-5D-3L (which is known to demonstrate ceiling effects), and that utility scores could be derived with a Dutch value set.^15,19^ The new cost estimates likely provide a better representation of the Dutch healthcare system in 2022, and also allowed for the assessment of cost-effectiveness from a societal perspective, as recommended by Dutch guidelines.^8^ As a societal perspective better encompasses the wider implications of providing late-window EVT, these results may further inform decision making on priority setting and resource allocation, especially in scenarios with workforce- or budget constraints.

Despite these strengths, this study also had several limitations. First, our analyses were performed from a Dutch cost perspective, which may not be generalisable to other settings. Second, cost- and utility estimates beyond 2 years post-stroke, as well as transition probabilities beyond 5 years post-stroke, were extrapolated from input parameters on earlier time points. The long-term transition probabilities were also not explicitly derived from an EVT-population. Still, synthesising input parameters from various sources and implementing these in a long-term model is the most cost- and data-efficient way of assessing lifetime cost-effectiveness. We also expect a limited impact of these factors on the reliability of the model, as the chosen input parameters were derived from cohorts closely resembling our model’s population, and an extensive PSA was performed.

The results of our present study suggest that late-window EVT in patients selected based on the presence of collaterals is a cost-effective strategy in The Netherlands and countries with comparable healthcare systems. As the MR CLEAN-LATE had very pragmatic and inclusive inclusion criteria, the results of this model-based cost-effectiveness analysis should be broadly generalisable to daily clinical practice. However, the current study does not encompass all patients currently eligible for late-window stroke treatment. Future studies may explore the overall cost-effectiveness of a combination of late-window EVT selection strategies using comprehensive real-world data, and identify specific subgroups in which EVT might not be (cost-)effective (e.g. based on pre-stroke disability or imaging characteristics). The latter point may be especially relevant, as the broad implementation of collateral-based selection for late-window EVT may entail substantial changes in hospital workload and resource allocation. As the results of our one-way sensitivity analysis suggest that uncertainty regarding the initial mRS distribution has the highest impact on cost-effectiveness, future studies might focus on deriving this data from larger cohorts. This may further advise clinicians and policy makers on priority setting under constraints.

## Conclusion

Collateral-based selection for late-window EVT is likely cost-effective compared to BMM from a Dutch societal perspective. These results may inform decision making on the implementation and reimbursement of this expansion of treatment criteria. Future studies may focus on using real-world data to assess the generalisability of our findings to populations outside of the scope of the MR CLEAN-LATE trial and identifying patients in which late-window EVT might not be (cost-)effective. As our analyses suggest that uncertainty regarding the mRS distribution at 3 months post-stroke has the highest impact on cost-effectiveness, this will be a key item to consider in future cost-effectiveness analyses.

## Supplemental Material

sj-docx-1-eso-10.1177_23969873231220464 – Supplemental material for Cost-effectiveness of endovascular treatment after 6–24 h in ischaemic stroke patients with collateral flow on CT-angiography: A model-based economic evaluation of the MR CLEAN-LATE trial Supplemental material, sj-docx-1-eso-10.1177_23969873231220464 for Cost-effectiveness of endovascular treatment after 6–24 h in ischaemic stroke patients with collateral flow on CT-angiography: A model-based economic evaluation of the MR CLEAN-LATE trial by Florentina ME Pinckaers, Silvia MAA Evers, Susanne GH Olthuis, Hieronymus D Boogaarts, Alida A Postma, Robert J van Oostenbrugge, Wim H van Zwam and Janneke PC Grutters in European Stroke Journal

sj-docx-2-eso-10.1177_23969873231220464 – Supplemental material for Cost-effectiveness of endovascular treatment after 6–24 h in ischaemic stroke patients with collateral flow on CT-angiography: A model-based economic evaluation of the MR CLEAN-LATE trial Supplemental material, sj-docx-2-eso-10.1177_23969873231220464 for Cost-effectiveness of endovascular treatment after 6–24 h in ischaemic stroke patients with collateral flow on CT-angiography: A model-based economic evaluation of the MR CLEAN-LATE trial by Florentina ME Pinckaers, Silvia MAA Evers, Susanne GH Olthuis, Hieronymus D Boogaarts, Alida A Postma, Robert J van Oostenbrugge, Wim H van Zwam and Janneke PC Grutters in European Stroke Journal
