# Supplementary material for: Cost-effectiveness of endovascular treatment after 6–24 h in ischaemic stroke patients with collateral flow on CT-angiography: A model-based economic evaluation of the MR CLEAN-LATE trial
Source: Eur Stroke J. 2023 Dec 28;9(2):348–55. doi: 10.1177/23969873231220464 (PMC11318439; doi:10.1177/23969873231220464)
Supplement: sj-docx-1-eso-10.1177_23969873231220464 – Supplemental material for Cost-effectiveness of endovascular treatment after 6–24 h in ischaemic stroke patients with collateral flow on CT-angiography: A model-based economic evaluation of the MR CLEAN-LATE trial [file sj-docx-1-eso-10.1177_23969873231220464.docx]

Supplemental Appendix

Cost-effectiveness of endovascular treatment after 6-24h in ischemic stroke patients with collateral flow on CT-angiography: a model-based economic evaluation of the MR CLEAN-LATE trial

Florentina M.E. Pinckaers, MD, Silvia M.A.A. Evers, PhD, Susanne G.H. Olthuis, MD, Hieronymus D. Boogaarts, MD, PhD, Alida A. Postma, MD, PhD, Robert J. van Oostenbrugge, MD, PhD, Wim H. van Zwam, MD, PhD, Janneke P.C. Grutters, PhD

Table of Contents

[Appendix I: Input parameters 2](#_Toc151572183)

[Appendix II: Cohort trace 11](#_Toc151572184)

[Appendix III: Formulas 12](#_Toc151572185)

[Appendix IV: ICER plot healthcare perspective 13](#_Toc151572186)

[Appendix V: One-way sensitivity analysis 14](#_Toc151572187)

[Appendix VI: Undiscounted results 15](#_Toc151572188)

[Appendix VII: MR CLEAN-LATE trial investigators and trial organization 16](#_Toc151572189)

# Appendix I: Input parameters

| Table S1: Model inputs | | | | | |
| --- | --- | --- | --- | --- | --- |
|  | **Base-case value** | **Distribution** | **(*α***, *β)* | **Source** | |
| **Probabilities** | | | | |  |
| BMM |  |  |  |  |  |
| mRS 0 | 0.020 | Dirichlet | (5, 242) | ^1^ |  |
| mRS 1 | 0.138 | Dirichlet | (34, 213) | ^1^ |  |
| mRS 2 | 0.182 | Dirichlet | (45, 202) | ^1^ |  |
| mRS 3 | 0.077 | Dirichlet | (19, 228) | ^1^ |  |
| mRS 4 | 0.113 | Dirichlet | (28, 219) | ^1^ |  |
| mRS 5 | 0.170 | Dirichlet | (42, 205) | ^1^ |  |
| mRS 6 | 0.300 | Dirichlet | (74, 173) | ^1^ |  |
| EVT |  |  |  |  |  |
| mRS 0 | 0.090 | Dirichlet | (23, 232) | ^1^ |  |
| mRS 1 | 0.122 | Dirichlet | (31, 224) | ^1^ |  |
| mRS 2 | 0.180 | Dirichlet | (46, 209) | ^1^ |  |
| mRS 3 | 0.114 | Dirichlet | (29, 226) | ^1^ |  |
| mRS 4 | 0.118 | Dirichlet | (30, 225) | ^1^ |  |
| mRS 5 | 0.133 | Dirichlet | (34, 221) | ^1^ |  |
| mRS 6 | 0.243 | Dirichlet | (62, 193) | ^1^ |  |
| **Transition probabilities** | | | | |  |
| From 3 months to 12 months |  |  |  |  |  |
| From mRS 0 to |  |  |  |  |  |
| mRS 0 | 0.548 | Dirichlet | (75.1, 61.9) | ^11^ |  |
| mRS 1 | 0.359 | Dirichlet | (49.2, 87.8) | ^11^ |  |
| mRS 2 | 0.064 | Dirichlet | (8.7, 128.3) | ^11^ |  |
| mRS 3 | 0.014 | Dirichlet | (2.0, 135.0) | ^11^ |  |
| mRS 4 | 0.000 | Dirichlet | (0.0, 137.0) | ^11^ |  |
| mRS 5 | 0.000 | Dirichlet | (0.0, 137.0) | ^11^ |  |
| mRS 6 | 0.014 | Dirichlet | (2.0, 135.0) | ^11^ |  |
| From mRS 1 to |  |  |  |  |  |
| mRS 0 | 0.143 | Dirichlet | (61.2, 365.8) | ^11^ |  |
| mRS 1 | 0.618 | Dirichlet | (263.8, 163.2) | ^11^ |  |
| mRS 2 | 0.163 | Dirichlet | (69.6, 357.4) | ^11^ |  |
| mRS 3 | 0.041 | Dirichlet | (17.6, 409.4) | ^11^ |  |
| mRS 4 | 0.009 | Dirichlet | (4.0, 423.0) | ^11^ |  |
| mRS 5 | 0.000 | Dirichlet | (0.0, 427.0) | ^11^ |  |
| mRS 6 | 0.025 | Dirichlet | (10.9, 416.1) | ^11^ |  |
| From mRS 2 to |  |  |  |  |  |
| mRS 0 | 0.070 | Dirichlet | (21.2, 283.8) | ^11^ |  |
| mRS 1 | 0.289 | Dirichlet | (88.1, 216.9) | ^11^ |  |
| mRS 2 | 0.455 | Dirichlet | (138.7, 166.3) | ^11^ |  |
| mRS 3 | 0.129 | Dirichlet | (39.2, 265.8) | ^11^ |  |
| mRS 4 | 0.029 | Dirichlet | (8.9, 296.1) | ^11^ |  |
| mRS 5 | 0.000 | Dirichlet | (0.0, 305.0) | ^11^ |  |
| mRS 6 | 0.029 | Dirichlet | (8.9, 296.1) | ^11^ |  |
| From mRS 3 to |  |  |  |  |  |
| mRS 0 | 0.000 | Dirichlet | (0.0, 251.0) | ^11^ |  |
| mRS 1 | 0.050 | Dirichlet | (12.7, 238.3) | ^11^ |  |
| mRS 2 | 0.164 | Dirichlet | (41.2, 209.8) | ^11^ |  |
| mRS 3 | 0.541 | Dirichlet | (135.7, 115.3) | ^11^ |  |
| mRS 4 | 0.123 | Dirichlet | (30.9, 220.1) | ^11^ |  |
| mRS 5 | 0.016 | Dirichlet | (4.0, 247.0) | ^11^ |  |
| mRS 6 | 0.106 | Dirichlet | (26.5, 224.5) | ^11^ |  |
| From mRS 4 to |  |  |  |  |  |
| mRS 0 | 0.000 | Dirichlet | (0.0, 180.0) | ^11^ |  |
| mRS 1 | 0.000 | Dirichlet | (0.0, 180.0) | ^11^ |  |
| mRS 2 | 0.038 | Dirichlet | (6.9, 173.1) | ^11^ |  |
| mRS 3 | 0.217 | Dirichlet | (39.0, 141.0) | ^11^ |  |
| mRS 4 | 0.482 | Dirichlet | (86.8, 93.2) | ^11^ |  |
| mRS 5 | 0.059 | Dirichlet | (10.7, 169.3) | ^11^ |  |
| mRS 6 | 0.204 | Dirichlet | (36.7, 143.3) | ^11^ |  |
| From mRS 5 to |  |  |  |  |  |
| mRS 0 | 0.000 | Dirichlet | (0.0, 103.0) | ^11^ |  |
| mRS 1 | 0.000 | Dirichlet | (0.0, 103.0) | ^11^ |  |
| mRS 2 | 0.000 | Dirichlet | (0.0, 103.0) | ^11^ |  |
| mRS 3 | 0.010 | Dirichlet | (1.0, 102.0) | ^11^ |  |
| mRS 4 | 0.136 | Dirichlet | (14.0, 89.0) | ^11^ |  |
| mRS 5 | 0.520 | Dirichlet | (53.6, 49.4) | ^11^ |  |
| mRS 6 | 0.335 | Dirichlet | (34.5, 68.5) | ^11^ |  |
| From 12 months onwards (yearly) |  |  |  |  |  |
| From mRS 0 to |  |  |  |  |  |
| mRS 0 | 0.865* | Dirichlet | (58.8, 9.2)* | ^11^ |  |
| mRS 1 | 0.091* | Dirichlet | (6.2, 61.8)* | ^11^ |  |
| mRS 2 | 0.018* | Dirichlet | (1.2, 66.8)* | ^11^ |  |
| mRS 3 | 0.000* | Dirichlet | (0.0, 68.0)* | ^11^ |  |
| mRS 4 | 0.000* | Dirichlet | (0.0, 68.0)* | ^11^ |  |
| mRS 5 | 0.000* | Dirichlet | (0.0, 68.0)* | ^11^ |  |
| mRS 6 | 0.025* | Dirichlet | (1.7, 66.3)* | ^11^ |  |
| From mRS 1 to |  |  |  |  |  |
| mRS 1 | 0.922* | Dirichlet | (271.9, 23.1)* | ^11^ |  |
| mRS 2 | 0.033* | Dirichlet | (9.6, 285.4)* | ^11^ |  |
| mRS 3 | 0.001* | Dirichlet | (0.2, 294.8)* | ^11^ |  |
| mRS 4 | 0.003* | Dirichlet | (0.7, 294.3)* | ^11^ |  |
| mRS 5 | 0.001* | Dirichlet | (0.2, 294.8)* | ^11^ |  |
| mRS 6 | 0.041* | Dirichlet | (12.2, 282.8)* | ^11^ |  |
| From mRS 2 to |  |  |  |  |  |
| mRS 2 | 0.921* | Dirichlet | (193.3, 16.7)* | ^11^ |  |
| mRS 3 | 0.004* | Dirichlet | (0.7, 209.3)* | ^11^ |  |
| mRS 4 | 0.001* | Dirichlet | (0.2, 209.8)* | ^11^ |  |
| mRS 5 | 0.001* | Dirichlet | (0.2, 209.8)* | ^11^ |  |
| mRS 6 | 0.073* | Dirichlet | (15.4, 194.6)* | ^11^ |  |
| From mRS 3 to |  |  |  |  |  |
| mRS 3 | 0.846* | Dirichlet | (167.5, 30.5)* | ^11^ |  |
| mRS 4 | 0.040* | Dirichlet | (7.8, 190.2)* | ^11^ |  |
| mRS 5 | 0.011* | Dirichlet | (2.2, 195.8)* | ^11^ |  |
| mRS 6 | 0.103* | Dirichlet | (20.4, 177.6)* | ^11^ |  |
| From mRS 4 to |  |  |  |  |  |
| mRS 4 | 0.856* | Dirichlet | (91.5, 15.5)* | ^11^ |  |
| mRS 5 | 0.012* | Dirichlet | (1.2, 105.8)* | ^11^ |  |
| mRS 6 | 0.133* | Dirichlet | (14.2, 92.8)* | ^11^ |  |
| From mRS 5 to |  |  |  |  |  |
| mRS 5 | 0.833* | Dirichlet | (40.0, 8.0)* | ^11^ |  |
| mRS 6 | 0.167* | Dirichlet | (8.0, 40.0)* | ^11^ |  |
| **Mortality penalty** | | | | |  |
| Age |  |  |  |  |  |
| 79 | 1.118 | - | - | ^13^ |  |
| 80 | 1.264 | - | - | ^13^ |  |
| 81 | 1.430 | - | - | ^13^ |  |
| 82 | 1.606 | - | - | ^13^ |  |
| 83 | 1.809 | - | - | ^13^ |  |
| 84 | 2.045 | - | - | ^13^ |  |
| 85 | 2.308 | - | - | ^13^ |  |
| 86 | 2.603 | - | - | ^13^ |  |
| 87 | 2.915 | - | - | ^13^ |  |
| 88 | 3.318 | - | - | ^13^ |  |
| 89 | 3.654 | - | - | ^13^ |  |
| 90 | 4.079 | - | - | ^13^ |  |
| 91 | 4.474 | - | - | ^13^ |  |
| 92 | 4.925 | - | - | ^13^ |  |
| 93 | 5.398 | - | - | ^13^ |  |
| 94 | 5.891 | - | - | ^13^ |  |
| **Costs (€)** | | | | |  |
| Healthcare perspective |  |  |  |  |  |
| First 3 months, no EVT |  |  |  |  |  |
| mRS 0 | 10756 | *γ* | (6, 1800) | ^14^ |  |
| mRS 1 | 8923 | *γ* | (33, 269) | ^14^ |  |
| mRS 2 | 11562 | *γ* | (55, 209) | ^14^ |  |
| mRS 3 | 21681 | *γ* | (26, 829) | ^14^ |  |
| mRS 4 | 34941 | *γ* | (32, 1102) | ^14^ |  |
| mRS 5 | 48308 | *γ* | (212, 228) | ^14^ |  |
| mRS 6 | 10234 | *γ* | (138, 74) | ^-^ |  |
| First 3 months, EVT |  |  |  |  |  |
| mRS 0 | 16888 | *γ* | (29, 577) | ^14^ |  |
| mRS 1 | 19655 | *γ* | (57, 344) | ^14^ |  |
| mRS 2 | 18676 | *γ* | (164, 114) | ^14^ |  |
| mRS 3 | 35730 | *γ* | (116, 307) | ^14^ |  |
| mRS 4 | 43494 | *γ* | (88, 493) | ^14^ |  |
| mRS 5 | 49159 | *γ* | (140, 351) | ^14^ |  |
| mRS 6 | 16276 | *γ* | (178, 91) | ^-^ |  |
| 3 months to 1 year |  |  |  |  |  |
| mRS 0 | 1701 | *γ* | (11, 149) | ^14^ |  |
| mRS 1 | 4569 | *γ* | (13, 349) | ^14^ |  |
| mRS 2 | 6072 | *γ* | (22, 279) | ^14^ |  |
| mRS 3 | 10263 | *γ* | (20, 503) | ^14^ |  |
| mRS 4 | 34479 | *γ* | (31, 1126) | ^14^ |  |
| mRS 5 | 45183 | *γ* | (52, 868) | ^14^ |  |
| mRS 6 | 0 | - | - |  |  |
| Year 2 |  |  |  |  |  |
| mRS 0 | 2804 | *γ* | (12, 242) | ^14^ |  |
| mRS 1 | 5436 | *γ* | (10, 516) | ^14^ |  |
| mRS 2 | 7948 | *γ* | (22, 360) | ^14^ |  |
| mRS 3 | 19364 | *γ* | (22, 855) | ^14^ |  |
| mRS 4 | 41170 | *γ* | (29, 1437) | ^14^ |  |
| mRS 5 | 60088 | *γ* | (44, 1351) | ^14^ |  |
| mRS 6 | 0 | *-* | - |  |  |
| Year 3 onwards (yearly) |  |  |  |  |  |
| mRS 0 | 3340 | *γ* | (12, 285) | ^14^ |  |
| mRS 1 | 4780 | *γ* | (8, 579) | ^14^ |  |
| mRS 2 | 7800 | *γ* | (22, 348) | ^14^ |  |
| mRS 3 | 25044 | *γ* | (24, 1043) | ^14^ |  |
| mRS 4 | 36368 | *γ* | (26, 1377) | ^14^ |  |
| mRS 5 | 59932 | *γ* | (38, 1560) | ^14^ |  |
| mRS 6 | 0 | *-* | - |  |  |
| Societal perspective |  |  |  |  |  |
| First 3 months, no EVT |  |  |  |  |  |
| mRS 0 | 15564 | *γ* | (11, 1461) | ^14^ |  |
| mRS 1 | 11943 | *γ* | (32, 373) | ^14^ |  |
| mRS 2 | 19417 | *γ* | (69, 282) | ^14^ |  |
| mRS 3 | 28099 | *γ* | (40, 699) | ^14^ |  |
| mRS 4 | 46948 | *γ* | (48, 982) | ^14^ |  |
| mRS 5 | 55957 | *γ* | (179, 312) | ^14^ |  |
| mRS 6 | 12805 | *-* | (216, 59) |  |  |
| First 3 months, EVT |  |  |  |  |  |
| mRS 0 | 17633 | *γ* | (30, 591) | ^14^ |  |
| mRS 1 | 21133 | *γ* | (58, 364) | ^14^ |  |
| mRS 2 | 27392 | *γ* | (161, 170) | ^14^ |  |
| mRS 3 | 45485 | *γ* | (128, 355) | ^14^ |  |
| mRS 4 | 51084 | *γ* | (151, 338) | ^14^ |  |
| mRS 5 | 55145 | *γ* | (139, 397) | ^14^ |  |
| mRS 6 | 18858 | *-* | (242, 78) |  |  |
| 3 months to 1 year |  |  |  |  |  |
| mRS 0 | 2655 | *γ* | (15, 179) | ^14^ |  |
| mRS 1 | 8133 | *γ* | (30, 268) | ^14^ |  |
| mRS 2 | 11256 | *γ* | (48, 233) | ^14^ |  |
| mRS 3 | 22695 | *γ* | (63, 362) | ^14^ |  |
| mRS 4 | 57741 | *γ* | (83, 695) | ^14^ |  |
| mRS 5 | 69645 | *γ* | (190, 367) | ^14^ |  |
| mRS 6 | 0 | - | - |  |  |
| Year 2 |  |  |  |  |  |
| mRS 0 | 5048 | *γ* | (10, 503) | ^14^ |  |
| mRS 1 | 9380 | *γ* | (21, 450) | ^14^ |  |
| mRS 2 | 15044 | *γ* | (52, 289) | ^14^ |  |
| mRS 3 | 40262 | *γ* | (65, 618) | ^14^ |  |
| mRS 4 | 70318 | *γ* | (82, 852) | ^14^ |  |
| mRS 5 | 92238 | *γ* | (99, 929) | ^14^ |  |
| mRS 6 | 0 | *-* | - |  |  |
| Year 3 onwards (yearly) |  |  |  |  |  |
| mRS 0 | 6620 | *γ* | (8, 783) | ^14^ |  |
| mRS 1 | 7916 | *γ* | (14, 579) | ^14^ |  |
| mRS 2 | 15080 | *γ* | (56, 267) | ^14^ |  |
| mRS 3 | 50264 | *γ* | (67, 753) | ^14^ |  |
| mRS 4 | 63645 | *γ* | (82, 778) | ^14^ |  |
| mRS 5 | 91616 | *γ* | (61, 1514) | ^14^ |  |
| mRS 6 | 0 | *-* | - |  |  |
| **Utilities** | | | | |  |
| First 3 months |  |  |  |  |  |
| mRS 0 | 0.240 | *γ* | (23104, 0.00003)** | ^14^ |  |
| mRS 1 | 0.225 | *γ* | (24025, 0.00003)** | ^14^ |  |
| mRS 2 | 0.192 | *γ* | (26082, 0.00003)** | ^14^ |  |
| mRS 3 | 0.140 | *γ* | (7396, 0.00012)** | ^14^ |  |
| mRS 4 | 0.088 | *γ* | (2719, 0.00034)** | ^14^ |  |
| mRS 5 | 0.045 | *γ* | (4053, 0.00024)** | ^14^ |  |
| mRS 6 | 0 | *-* | - |  |  |
| 3 months to 1 year |  |  |  |  |  |
| mRS 0 | 0.720 | *γ* | (1394, 0.00020)** | ^14^ |  |
| mRS 1 | 0.637 | *γ* | (584, 0.00062)** | ^14^ |  |
| mRS 2 | 0.578 | *γ* | (793, 0.00053)** | ^14^ |  |
| mRS 3 | 0.420 | *γ* | (239, 0.00242)** | ^14^ |  |
| mRS 4 | 0.292 | *γ* | (356, 0.00199)** | ^14^ |  |
| mRS 5 | 0.082 | *γ* | (599, 0.00153)** | ^14^ |  |
| mRS 6 | 0 | *-* | - |  |  |
| Year 2 |  |  |  |  |  |
| mRS 0 | 0.945 | *γ* | (13, 0.00409)** | ^14^ |  |
| mRS 1 | 0.860 | *γ* | (196, 0.00071)** | ^14^ |  |
| mRS 2 | 0.755 | *γ* | (267, 0.00092)** | ^14^ |  |
| mRS 3 | 0.535 | *γ* | (346, 0.00134)** | ^14^ |  |
| mRS 4 | 0.345 | *γ* | (350, 0.00187)** | ^14^ |  |
| mRS 5 | 0.090 | *γ* | (518, 0.00176)** | ^14^ |  |
| mRS 6 | 0 | *-* | - |  |  |
| Year 3 onwards (yearly) |  |  |  |  |  |
| mRS 0 | 0.930 | *γ* | (12, 0.00571)** | ^14^ |  |
| mRS 1 | 0.870 | *γ* | (169, 0.00077)** | ^14^ |  |
| mRS 2 | 0.740 | *γ* | (169, 0.00154)** | ^14^ |  |
| mRS 3 | 0.510 | *γ* | (150, 0.00327)** | ^14^ |  |
| mRS 4 | 0.300 | *γ* | (136, 0.00514)** | ^14^ |  |
| mRS 5 | 0.070 | *γ* | (177, 0.00527)** | ^14^ |  |
| mRS 6 | 0 | *-* | - |  |  |
| Abbreviations: BMM=best medical management; EVT=endovascular treatment; mRS=modified Rankin Scale; PSA=probabilistic sensitivity analysis.  *For transitions beyond 5-years post-stroke, the mortality probability was inflated using an age-dependent mortality penalty. Any increase in the probability of death was accompanied by a decrease in probability of remaining in the same health state. E.g.: if the mortality probability would increase with 0.05, the steady-state probability would be reduced with 0.05.  **As utilities were not constraint to positive values in our analyses, PSA parameters were derived using disutilities (1 - utility). | | | | |  |

# Appendix II: Cohort trace


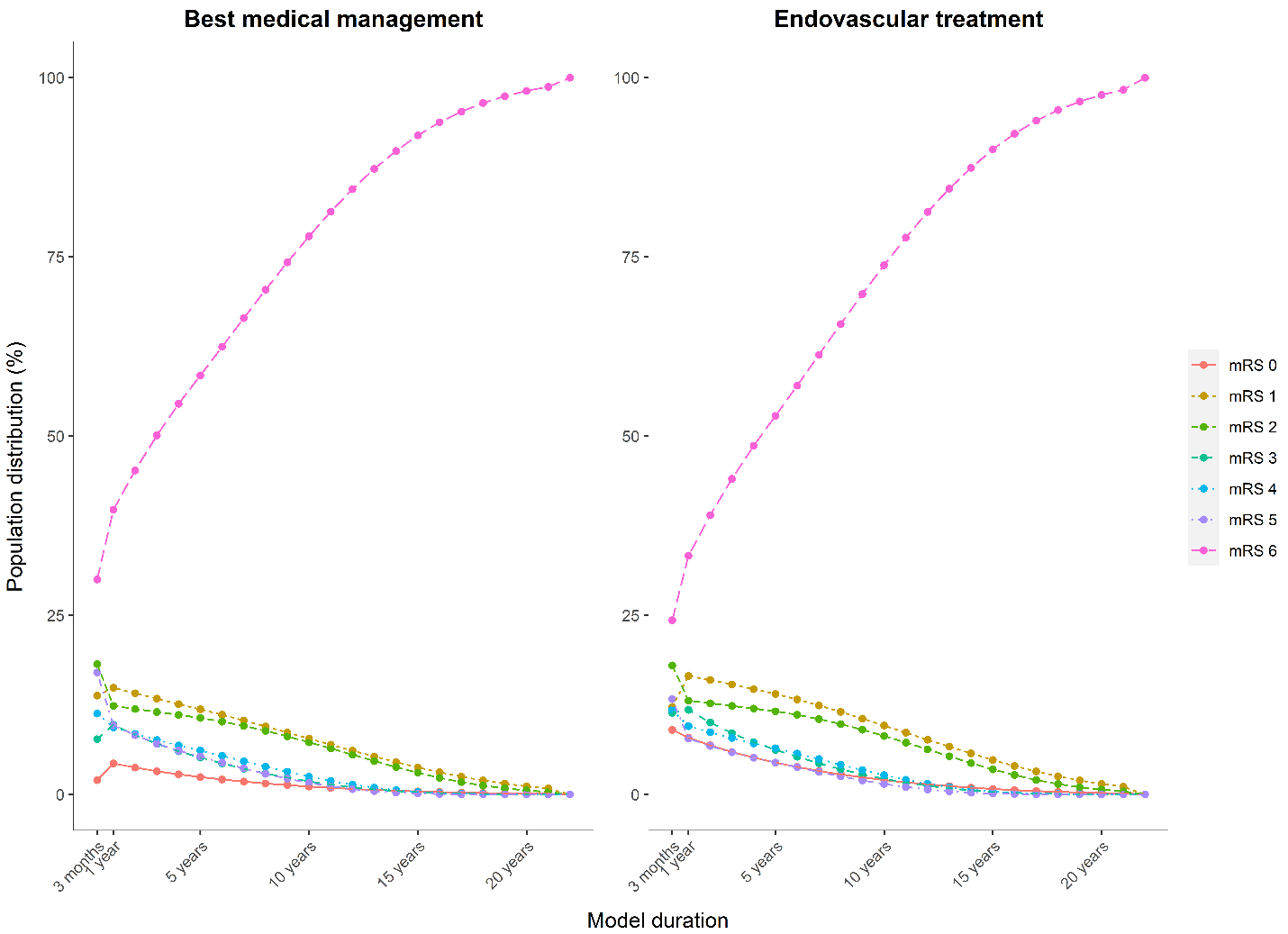
*Figure S1: Cohort trace plot.*

# Appendix III: Formulas

Incremental cost-effectiveness ratio (ICER):

$$ICER=\frac{\text{incremental costs}}{\text{incremental effects}}$$

Incremental net monetary benefit (NMB):

$$\text{incremental NMB}=\left( \text{incremental effects}\times\text{cost-effectiveness threshold} \right)-\text{incremental costs}$$

# Appendix IV: ICER plot healthcare perspective

*Figure S2: Results of the probabilistic sensitivity analysis (healthcare perspective). The dashed lines indicate the cost-effectiveness thresholds of €50,000 and €80,000 per QALY.*


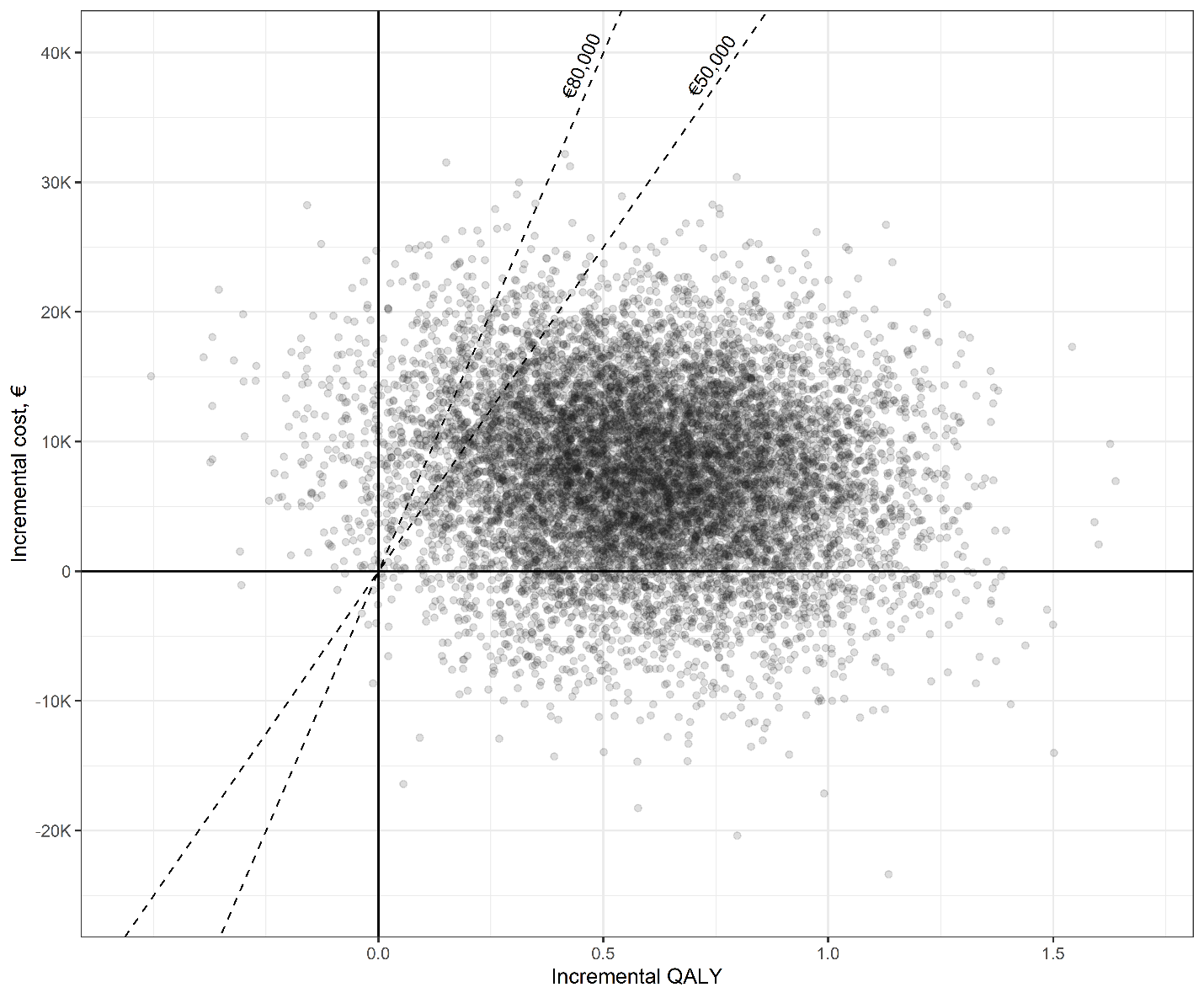


# Appendix V: One-way sensitivity analysis

*Figure S3: Tornado diagram of the one-way sensitivity analysis. Each bar depicts the effect of changing a single parameter on the iNMB. The upper and lower bounds were derived from the simulated PSA range. As the initial cohort distribution was varied using a Dirichlet distribution, the mRS distribution samples with the lowest and highest mortality probability were used.*


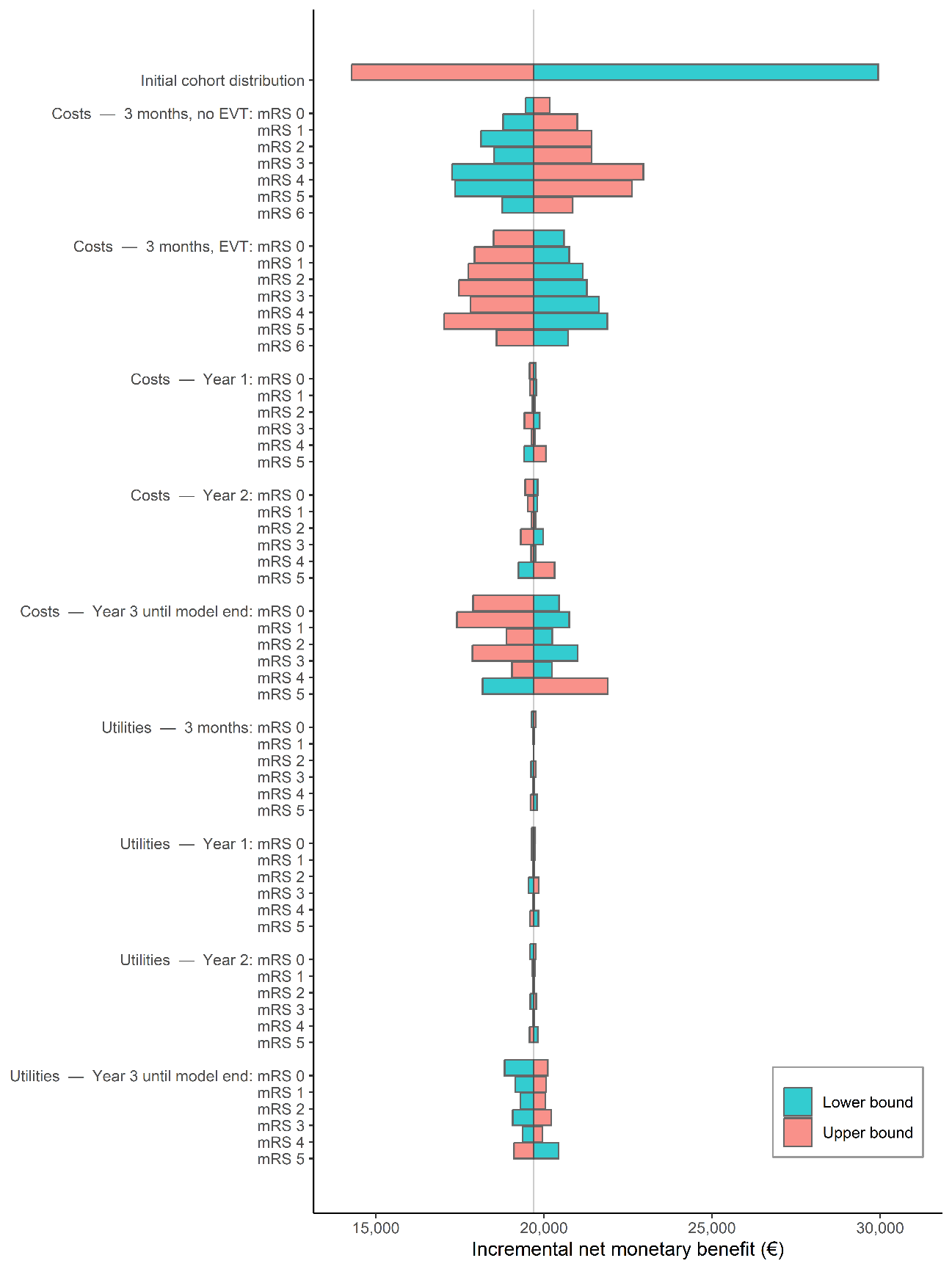


# Appendix VI: Undiscounted results

| Table S2: Undiscounted results from a societal perspective | | | | | | |
| --- | --- | --- | --- | --- | --- | --- |
| **Strategy** | **Costs, € (95%CI)** | **Effects, QALY (95%CI)** | **Incremental costs, € (95%CI)** | **Incremental effects, QALY (95%CI)** | **ICER, €/QALY** | **Incremental NMB, € (95%CI)*** |
| **Base case** | | | | | | |
| BMM | 211,471 | 3.92 | - | - | - | - |
| EVT | 226,006 | 4.70 | 14,535 | 0.78 | 18,565 | 24,612 |
| PSA | | | | | | |
| BMM | 212,144 (172,261 to 262,815) | 3.54 (2.96 to 4.20) | - | - | - | - |
| EVT | 226,548 (188,091 to 274,317) | 4.36 (3.69 to 5.08) | 14,404 (-14,383 to 42,101) | 0.82 (0.14 to 1.49) | 17,607 | 26,499 (-15,270 to 69,387) |
| *Abbreviations*: BMM=best medical management; EVT=endovascular treatment; ICER=incremental cost-effectiveness ratio; NMB=net monetary benefit; PSA=probabilistic sensitivity analysis; QALY=quality-adjusted life year.  *NMB at a willingness-to-pay threshold of €50,000/QALY. | | | | | | |

| Table S3: Undiscounted results from a healthcare perspective | | | | | | |
| --- | --- | --- | --- | --- | --- | --- |
| **Strategy** | **Costs, € (95%CI)** | **Effects, QALY (95%CI)** | **Incremental costs, € (95%CI)** | **Incremental effects, QALY (95%CI)** | **ICER, €/QALY** | **Incremental NMB, € (95%CI)*** |
| **Base case** | | | | | | |
| BMM | 127,798 | 3.92 | - | - | - | - |
| EVT | 137,927 | 4.70 | 10,129 | 0.78 | 12,937 | 29,018 |
| **PSA** | | | | | | |
| BMM | 128,503 (102,257 to 162,833) | 3.54 (2.96 to 4.20) | - | - | - | - |
| EVT | 138,484 (112,810 to 170,601) | 4.36 (3.69 to 5.08) | 9,981 (-8,218 to 27,466) | 0.82 (0.14 to 1.49) | 12,201 | 30,922 (-7,176 to 69,348) |
| *Abbreviations*: BMM=best medical management; EVT=endovascular treatment; ICER=incremental cost-effectiveness ratio; NMB=net monetary benefit; PSA=probabilistic sensitivity analysis; QALY=quality-adjusted life year.  *NMB at a willingness-to-pay threshold of €50,000/QALY. | | | | | | |

# Appendix VII: MR CLEAN-LATE trial investigators and trial organization

**The MR CLEAN-LATE investigators**

*Principal investigators*
Robert J. van Oostenbrugge (MD, PhD)^1,2^, Wim H. van Zwam (MD, PhD)^2,3^

*Study Coordinators*
Susanne G.H. Olthuis (MD)^1,2^, F.A.V. (Anne) Pirson (MD, PhD)^1,2^, Wouter H. Hinsenveld (MD)^1,2^, Robert-Jan B. Goldhoorn (MD, PhD)^1,2^

*Local principal investigators*
Robert J. van Oostenbrugge (MD, PhD)^1,2^, Wim H. van Zwam (MD, PhD)^1,2^, Julie Staals (MD, PhD)^1,2^, Diederik W.J. Dippel (MD, PhD)^4^, Aad van der Lugt (MD, PhD)^5^, Adriaan C.G.M. van Es (MD, PhD)^6^, Bob Roozenbeek (MD, PhD)^4^, Pieter-Jan van Doormaal, Yvo B.W.E.M. Roos (MD, PhD)^7^, Charles B.L.M. Majoie (MD, PhD)^8^, Jonathan M. Coutinho (MD, PhD)^7^, Bart J. Emmer (MD, PhD)^8^, H Bart van der Worp (MD, PhD)^9^, Rob T.H. Lo (MD, PhD)^10^, Marianne A.A. van Walderveen (MD, PhD)^6^, Marieke J.H. Wermer (MD, PhD)^11^, Ewoud I. van Dijk (MD, PhD)^12^, Sjoerd Jenniskens (MD)^13^, H.D. (Jeroen) Boogaarts (MD, PhD)^14^, Maarten Uyttenboogaart (MD, PhD)^15^, Reinoud P.H. Bokkers (MD, PhD)^16^, Koos Keizer (MD, PhD)^17^, Rob A.R. Gons (MD, PhD)^17^, Lonneke S.F. Yo (MD)^18^, Heleen M. den Hertog (MD, PhD)^19^, Boudewijn A.A.M. van Hasselt (MD)^20^, Wouter J. Schonewille (MD, PhD)^21^, Jan-Albert Vos (MD, PhD)^22^, Julia H. van Tuijl (MD, PhD)^23^, Issam Boukrab (MD)^24^, Hans G. Kortman (MD)^24^, Jeannette Hofmeijer (MD, PhD)^25,26^, Jasper M. Martens (MD)^27^, Ido R. van den Wijngaard (MD, PhD)^11,28^, Jelis Boiten (MD, PhD)^28^, Geert J. Lycklama à Nijeholt (MD, PhD)^29^, Paul J.A.M. Brouwers (MD, PhD)^30^, Emiel J.C. Sturm (MD)^31^, Tomas Bulut (MD)^31^, Karlijn F. de Laat (MD, PhD)^32^, Lukas C. van Dijk (MD, PhD)^33^, Michel J.M. Remmers (MD)^34^, Thijs E.A.M. de Jong (MD)^35^, Anouk D. Rozeman (MD, PhD)^36^, Otto E.H. Elgersma (MD)^37^, Bas van der Veen (MD)^38^, Davy R. Sudiono (MD)^39^

**Trial collaborators**

*Executive committee*
Robert J. van Oostenbrugge (MD, PhD)^1,2^, Wim H. van Zwam (MD, PhD)^2,3^, Marianne A.A. van Walderveen (MD, PhD)^6^, Geert J. Lycklama à Nijeholt (MD, PhD)^29^, Wouter J. Schonewille (MD, PhD)^21^, Maarten Uyttenboogaart (MD, PhD)^15^, Charles B.L.M. Majoie (MD, PhD)^8^

*Data monitoring committee*
Heinrich Mattle (MD, PhD)^40^ – *Chair*, professor of Neurology; Jens Fiehler (MD, PhD)^41^, professor of Interventional Neuroradiology; Sander van Kuijk (PhD)^42^, independent statistician

*Independent trial statistician*
Daan Nieboer (MSc)^43^

*Statistical advisor and trial methodologist*
Hester F. Lingsma (PhD)^43^

**Contrast Clinical trial collaborators**

*Research leaders*
Diederik W.J. Dippel (MD, PhD)^4^, Charles B.L.M. Majoie (MD, PhD)^8^

*Consortium coordinator*
Rick van Nuland (PhD)^44^

*Imaging assessment committee*
Charles B.L.M. Majoie (MD, PhD)^8^ – *Chair*, Aad van der Lugt (MD, PhD)^5^ – *Chair*, Wim H. van Zwam (MD, PhD)^2,3^, Bart J. Emmer (MD, PhD)^8^, Stefan D. Roosendaal (MD, PhD)^8^, Lonneke S.F. Yo (MD)^18^, G.M. (Menno) Krietemeijer (MD)^18^, Adriaan C.G.M. van Es (MD, PhD)^6^, Pieter-Jan van Doormaal (MD)^5^, Alida A. Postma (MD, PhD)^3,45^, Geert J. Lycklama à Nijeholt (MD, PhD)^29^, René van den Berg (MD, PhD)^8^, Ludo Beenen (MD. PhD)^8^, Jasper M. Martens (MD)^27^, Sebastiaan Hammer (MD, PhD)^33^, Anton Meijer (MD, PhD)^13^, Reinoud P.H. Bokkers (MD, PhD)^16^, Anouk van der Hoorn (MD, PhD)^16^, Ido R. van den Wijngaard (MD, PhD)^28,11^, Albert J. Yoo (MD, PhD)^46^, Dick Gerrits (MD)^31^

*Adverse event committee*
Robert J. van Oostenbrugge (MD, PhD)^1,2^ – *Chair*, Bart J. Emmer (MD, PhD)^8^, Jonathan M. Coutinho (MD, PhD)^7^, Ben P.W. Jansen (MD)^23^ , Martine T.B. Truijman (MD, PhD)^1^, Julie Staals (MD, PhD)^1,2^

*Outcome assessment committee*
Yvo B.W.E.M. Roos (MD, PhD)^7^ – *Chair*, Sanne M. Manschot (MD, PhD)^28^, Diederik W.J. Dippel (MD, PhD)^4^, Henk Kerkhoff (MD, PhD)^36^, Ido R. van den Wijngaard (MD, PhD)^11,28^, Jonathan M. Coutinho (MD, PhD)^7^, Peter J. Koudstaal (MD, PhD)^4^, Koos Keizer (MD, PhD)^17^, Jelis Boiten (MD, PhD)^28^

*Data management group*
Hester F. Lingsma (PhD)^43^, Diederik W.J. Dippel (MD, PhD)^4^, Vicky Chalos (MD)^4^, Olvert A. Berkhemer (MD, PhD)^8^

*Imaging data management*
Aad van der Lugt (MD, PhD)^5^, Charles B.L.M. Majoie (MD, PhD)^8^, Adriaan Versteeg (BSc)^5^, Lennard Wolff (MD)^5^, Jiahang Su (MSc)^5^, P.M. (Matthijs) van der Sluijs (MD)^5^, Henk van Voorst (MD)^8,47^, Manon Tolhuisen (MSc)^8^

*Biomaterials and translational group*
Hugo ten Cate (MD, PhD)^2,48,49,50^, Moniek P.M. de Maat (PhD)^51^, Samantha Donse-Donkel (MD)^51^, Heleen M.M. van Beusekom (PhD)^52^, Aladdin Taha (MD)^4,52^, Aarazo Barakzie (MSc)^51^

*Local collaborators*
Kilian Treurniet (MD, PhD)^8^, Sophie van den Berg (MD)^7^, Natalie LeCouffe (MD)^7^, Rob van de Graaf (MD, PhD)^5^, Robert-Jan Goldhoorn (MD, PhD)^1,2^, Inger R. de Ridder (MD, PhD)^1,2^, Wouter H. Hinsenveld (MD)^1,2^, F.A.V. (Anne) Pirson (MD, PhD)^1,2^, Susanne G.H. Olthuis (MD)^1,2^, Florentina M.E. Pinckaers (MD)^2,3^, Angelique Ceulemans (MSc)^1,2^, Robrecht R.M.M. Knapen (MD)^2,3^, M.M.Q. (Quirien) Robbe (MD)^2,3^, Lotte Sondag (MD)^12^, Manon Kappelhof (MD, PhD)^8^, Rik Reinink (MD)^9^, Suzanne M. Silvis (MD, PhD)^36^, Floris H.B.M. Schreuder (MD, PhD)^12^, Simone Uniken Venema (MD)^9^, Laura C.C. van Meenen (MD, PhD)^7^, Sabine Collette (MD)^15,16^, Wilma van Wijngaarden^21^, Wouter van der Steen (MD)^4^, Jan W. Hoving (MD)^8^

*Research nurses*
Sabrina P.J.H. Verheesen (BSc)^1^, Martin Sterrenberg^4^, Naziha El Ghannouti^4^, Rita Sprengers^7^, Ayla van Ahee^9^, Berber Zweedijk^9^, Wilma Pellikaan^21^, Irati Schonewille^21^, Kitty Blauwendraat^21^, Yvonne Drabbe^32^, Anke Kleine-Kathöfer^32^, Joke de Meris^28^, Michelle Sandiman^32^, Tamara Dofferhoff-Vermeulen^28^, Michelle Simons^25^, Hester Bongenaar^17^, Maylee Smallegange^17^, Anja van Loon^34^, Karin Kraus^34^, Erna Bos-Verheij^11^, Esther Santegoets^23^, Suze Kooij (BSc)^37^, Annemarie Slotboom^36^, Eva Ponjee^19^, Rieke Eilander^19^, Hanneke Droste^30^, Esther van Veen^30^, Rosalie Visser^30^, Jasmijn Lodico^30^, Marieke de Jong^15^, Friedus van der Minne^15^, Eefje Cleophas (MA)^1^, Ernst B. Muskens^15^, Amy Nijst (Msc)^32^

*Study monitors*
Leontien Heiligers^5^, Naziha El Ghannouti^4^, Yvonne Martens (MSc)^5^, Miranda Slotboom^5^

*Independent expert*
Rogier G. Hintzen (MD, PhD)^4^, Bart C. Jacobs (MD, PhD)^4,53^

**Affiliations**

^1^ Department of Neurology, Maastricht University Medical Center+, Maastricht, The Netherlands;
^2^ School for Cardiovascular Diseases (CARIM), Maastricht University, Maastricht, The Netherlands;
^3^ Department of Radiology and Nuclear Medicine, Maastricht University Medical Center+, Maastricht, The Netherlands;
^4^ Department of Neurology, Erasmus MC, University Medical Center Rotterdam, Rotterdam, The Netherlands;
^5^ Department of Radiology and Nuclear Medicine, Erasmus MC, University Medical Center Rotterdam, Rotterdam, The Netherlands;
^6^ Department of Radiology, Leiden University Medical Center, Leiden, The Netherlands;
^7^ Department of Neurology, Amsterdam UMC location University of Amsterdam, Amsterdam, The Netherlands;
^8^ Department of Radiology and Nuclear Medicine, Amsterdam UMC location University of Amsterdam, Amsterdam, The Netherlands;
^9^ Department of Neurology and Neurosurgery, Brain Center, University Medical Center Utrecht, Utrecht, the Netherlands;
^10^ Department of Radiology, University Medical Center Utrecht, Utrecht, the Netherlands;
^11^ Department of Neurology, Leiden University Medical Center, Leiden, The Netherlands;
^12^ Department of Neurology, Radboud University Medical Center, Nijmegen, the Netherlands;
^13^ Department of Radiology, Radboud University Medical Center, Nijmegen, the Netherlands;
^14^ Department of Neurosurgery, Radboud University Medical Center, Nijmegen, the Netherlands;
^15^ Department of Neurology, University Medical Center Groningen, University of Groningen, Groningen, The Netherlands;
^16^ Department of Radiology, University Medical Center Groningen, University of Groningen, Groningen, The Netherlands;
^17^ Department of Neurology, Catharina Hospital, Eindhoven, The Netherlands;
^18^ Department of Radiology, Catharina Hospital, Eindhoven, The Netherlands;
^19^ Department of Neurology, Isala Hospital, Zwolle, The Netherlands;
^20^ Department of Radiology, Isala Hospital, Zwolle, The Netherlands;
^21^ Department of Neurology, Sint Antonius Hospital, Nieuwegein, The Netherlands;
^22^ Department of Radiology, Sint Antonius Hospital, Nieuwegein, The Netherlands;
^23^ Department of Neurology, Elisabeth-TweeSteden Hospital, Tilburg, The Netherlands;
^24^ Department of Radiology, Elisabeth-TweeSteden Hospital, Tilburg, The Netherlands;
^25^ Department of Neurology, Rijnstate Hospital, Arnhem, The Netherlands;
^26^ Department of Clinical Neurophysiology, University of Twente, Enschede, The Netherlands;
^27^ Department of Radiology and Nuclear Medicine, Rijnstate Hospital, Arnhem, The Netherlands;
^28^ Department of Neurology, Haaglanden Medical Center, the Hague, The Netherlands;
^29^ Department of Radiology, Haaglanden Medical Center, The Hague, The Netherlands;
^30^ Department of Neurology, Medisch Spectrum Twente, Enschede, The Netherlands;
^31^ Department of Radiology, Medisch Spectrum Twente, Enschede, The Netherlands;
^32^ Department of Neurology, HagaZiekenhuis, the Hague, The Netherlands;
^33^ Department of Radiology, HagaZiekenhuis, the Hague, The Netherlands;
^34^ Department of Neurology, Amphia Hospital, Breda, The Netherlands;
^35^ Department of Radiology, Amphia Hospital, Breda, The Netherlands;
^36^ Department of Neurology, Albert Schweitzer Hospital, Dordrecht, The Netherlands;
^37^ Department of Radiology, Albert Schweitzer Hospital, Dordrecht, The Netherlands;
^38^ Department of Neurology, Noordwest ziekenhuisgroep, Alkmaar, The Netherlands;
^39^ Department of Radiology, Noordwest ziekenhuisgroep, Alkmaar, The Netherlands;
^40^ University Clinic for Neurology, Inselspital, Bern, Switzerland;
^41^ Department of Radiology, University of Hamburg, Germany;
^42^ Department of Clinical Epidemiology and Medical Technology Assessment, Maastricht University Medical Center+, Maastricht, The Netherlands;
^43^ Department of Public Health, Erasmus MC, University Medical Center Rotterdam, Rotterdam, The Netherlands;
^44^ Lygature, Utrecht, The Netherlands;
^45^ School for Mental Health and Sciences (MHeNS), Maastricht university, Maastricht, The Netherlands;
^46^ Texas Stroke Institute, Plano, United States of America;
^47^ Department of Biomedical Engineering and Physics, Amsterdam UMC, University of Amsterdam, Amsterdam, The Netherlands;
^48^ Department of biochemistry and internal medicine, Maastricht University Medical Center+, Maastricht, The Netherlands;
^49^ Thrombosis Expertise Center, Heart & Vascular Center, Maastricht University Medical Center+, Maastricht, The Netherlands;
^50^ Center for Thrombosis and Hemostasis (CTH), University Medical Center of the Johannes Gutenberg University Mainz, Mainz, Germany;
^51^ Department of Hematology, Erasmus MC, University Medical Center Rotterdam, Rotterdam, The Netherlands;
^52^ Department of Cardiology, Erasmus MC, University Medical Center Rotterdam, Rotterdam, The Netherlands;
^53^ Department of Immunology, Erasmus MC, University Medical Center Rotterdam, Rotterdam, The Netherlands.
